# Awareness of complications and maintenance mode of oral piercing in a group of adolescents and young Italian adults with intraoral piercing

**DOI:** 10.4317/medoral.20487

**Published:** 2015-04-10

**Authors:** Iole Vozza, Francesca Fusco, Denise Corridore, Livia Ottolenghi

**Affiliations:** 1 Oral and Maxillo-facial Sciences Department, Sapienza University of Rome, Rome, Italy

## Abstract

**Background:**

The aim of the study was to focus the awareness of complications of oral piercing among a group of adolescents and young Italian adults with intraoral piercings.

**Material and Methods:**

A total of 225 teenagers were asked to complete a questionnaire on the awareness of complications of oral piercing. An additional questionnaire was administered in case of oral piercing worn, based on site piercing, knowledge about piercer license, oral and systemic risks due to oral piercing, disinfection and sterilization of the material pierced, information by the piercer about piercing hygiene maintenance and post-piercing dentist check-up. After questionnaire all partecipants received a brochure with some information about risks and maintenance mode of piercing.

**Results:**

Data revealed that more than 50% of teens surveyed was found to wear a piercing. Only 25.3% was aware of the risk of HCV cross-infection and only 17.3% reported of knowledge about risk of endocarditis. Only 17% checked the piercer license and only 18% sterilization and disinfection of the materials used. 53.7% did not received explanations about the risks associated with piercing. With regard to the maintenance mode of the piercing, it has been suggested to brush the piercing bar in 17% of cases. The post piercing specialist visits have been suggested only in 7% of cases.

**Conclusions:**

The general lack of awareness of complications and maintenance mode related to oral piercing needs to be addressed by some education programs performed at school and by dentists.

**Key words:** Oral piercing, oral health, oral complications.

## Introduction

In antiquity, body piercing was a cultural practice used in ceremonial or religious rites. Today, it has become popular amongst adolescents and young adults as a method of self-expression (1,2). Common sites for body piercing are ear lobes, noses, eyebrows, navels, nipples, and the genitals. However, piercing of the lips, cheeks, tongue, uvula, or a combination of these sites is of strict interest to the dental profession (3). The tongue is the most commonly pierced oral site. Different complications and side effects are associated with intraoral piercing, including pain, swelling, infection, gingival trauma, gingival recession, chipped or fractured teeth, increased salivary flow, metal hypersensitivity, and interference with speech and swallowing (4).Then it was found that tongue piercing may result in the colonization of periodontopathogenic bacteria at the piercing site in the absence of appropriate oral hygiene practices (5). It can also lead to airway obstruction (2) and midline diastema (6). Systemically, oral piercing has also been identified as a possible vector for the transmission of blood-borne viruses, such as human immunodeficiency virus (HIV), hepatitis (HAV, HBV, HCV), herpes simplex (HSV), and the Epstein-Barr virus (EBV). Furthermore, oral piercing may cause bacterial pathologies, such as Neisseria-induced endocarditis, Streptococcus viridans endocarditis, and Ludwig angina (7). Late complications can lead to bifid tongue, atypical trigeminal neuralgia, lesion of soft tongue tissue, hypertrophic-keloid lesion (7). Adding to the concern of possible complication of intraoral piercing is the level of awareness of these complications. Levin *et al*. reported a general lack of awareness of the complications of intraoral piercing (57.8%) in a group of 389 young Israeli adults (8). Gallè *et al*, found that on 3,868 young adults tested 84.4% declared to know the infectious risks associated with body piercing, but only 4.1% of them correctly identified the infectious diseases which can be transmitted through these procedures; while 59.2% of the sample declared that noninfectious diseases can occur after a tattoo or a piercing, but only 5.4% of them correctly identified them (9). The aim of the present study were to determine the awareness of complications of oral piercing in a group of adolescents and young Italian adults with intraoral piercing.

## Material and Methods

The present study was carried out at five high schools of Latina, Italy, and included 225 (125 males and 100 females) adolescents and young adults in the age group of 14-22 years. They were chosen randomly and exactly 45 students per high school. The ethical clearance was granted by the Research and Ethical Committee of Sapienza University of Rome. They were asked to complete a questionnaire as accurately as possible to collect data on eventual type of piercing worn, and awareness of complications of oral piercing . The variables related to the knowledge of oral piercing complications were: HIV, HAV, HBV, HCV, HSV infection; temporary paralysis, permanent paralysis, endocarditis, Ludwig Angina, allergic reactions to materials, gingival infections, gingival recession, chipped or fractured tooth, diastema, hyper salivation, lingual abscess. An additional questionnaire was administered in case of oral piercing worn, based on site piercing (lips, tongue, labial or lingual frenulum, cheeks); knowledge about piercer license, oral and systemic risks due to oral piercing, disinfection and sterilization of the material pierced; information by the piercer about piercing hygiene maintenance( rinses with mouthwash, brushing the bar piercing, using of antibiotic creams, post-piercing dental visit). Names were not being recorded on the questionnaire, to ensure anonymity. All participants in this study received a consent form and a cover letter explaining the study. The information reached from the questionnaires was captured in an electronic database, which was verified and validated. The answers to the questions were summarized by calculating the percentages of responses in the respective categories.

## Results

**Figure 1:**
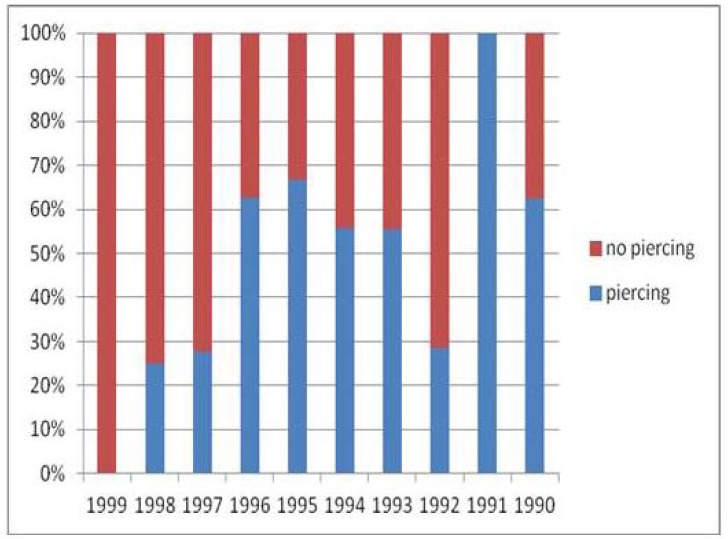
Percentage frequency of oral piercings by year of birth.

**Figure 2:**
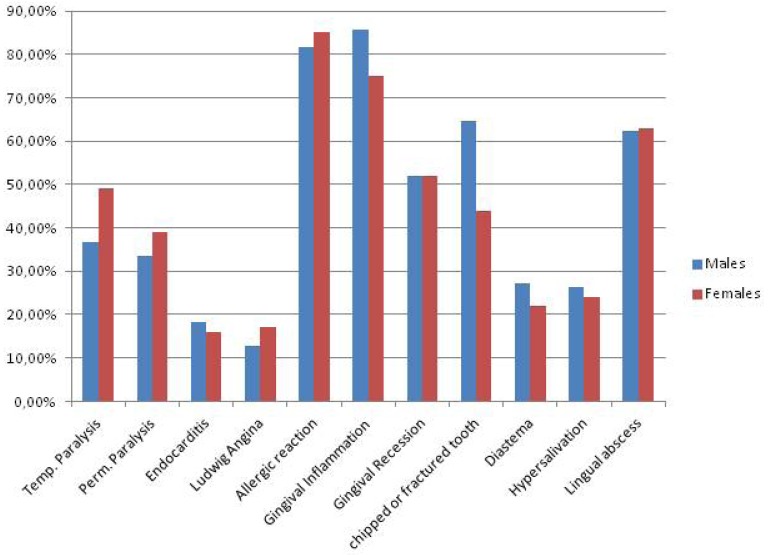
Awareness of the complications of oral piercing. Differences in male / female.

**Figure 3:**
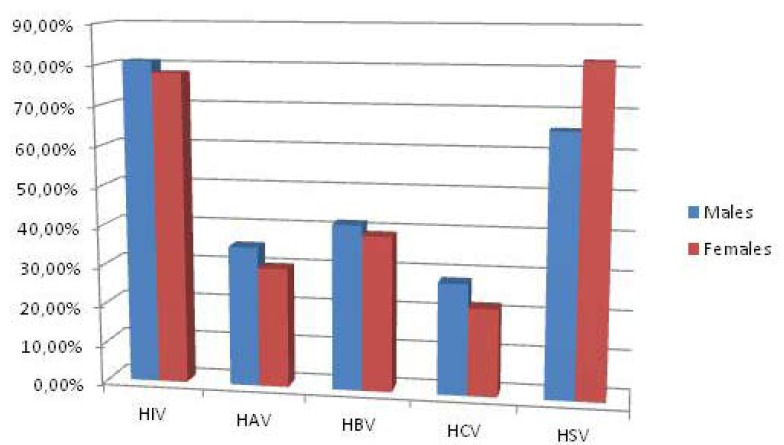
Awareness of viral complications. Differences in male / female.

**Figure 4:**
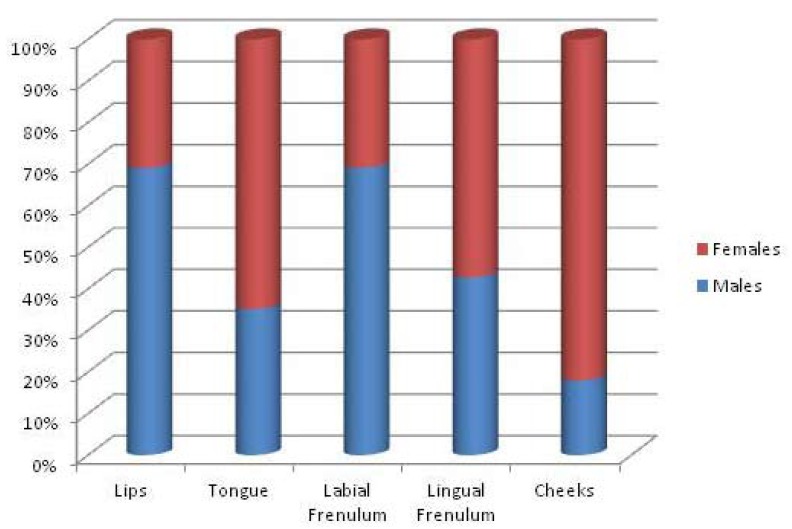
Oral piercing site, differences males / females.

**Figure 5:**
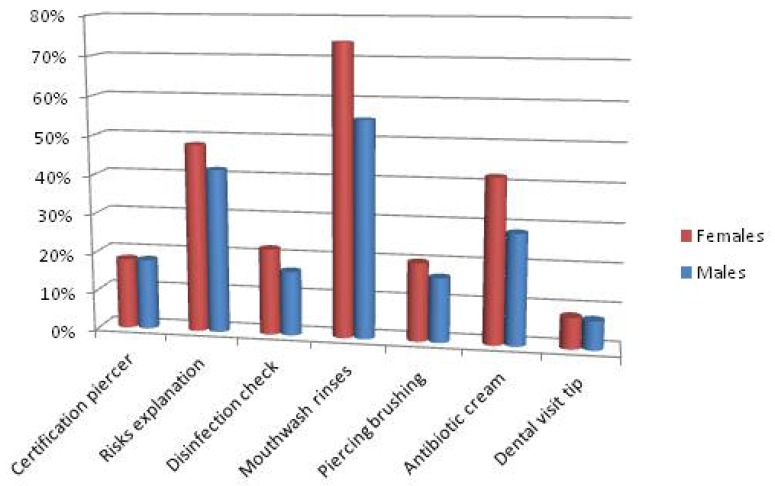
Information regarding the application of oral piercing. Differences in male / female.

The results of the present investigation are summarized in (Figs. 1-5).

Piercing distribution in relation to the year of birth is shown in figure 1. With the exception of the youngest, born in 1999, all age groups had a high percentage of piercing holder, which was under 30% for those born in the years 1998, 1997 and 1992. In the other cases the percentage exceeded 50% of the respondents. All born in 1991 (n = 3) said to wear an oral piercing (Fig. 1).

The awareness expressed by all the students interviewed about problems of cross-infection and piercing complications was more diffused for some issues than others. 79.5% (n . 179) showed knowledge about the risk of HIV infection and 72.8% (n. 164) for Herpes Simplex Virus, while the percentage decreased significantly with regard to hepatitis viruses (32.8% [n. 74] for HAV, 40.4% [n. 91] for HBV and 25.3% [n. 57] for HCV). However awareness of risks to overall health was poor. In fact, only 39 people reported of knowledge about risk of endocarditis (17.3%) and 33 about Ludwig Angina (14.6%). Knowledge about risk of temporary paralysis was found in 95 people (42.2%) and about permanent paralysis in 81 students (36%). About allergic reactions to materials used and gingival infections awareness was higher than 80% (187 people responded positively). About half of the respondents were aware of the risk of tooth chipping or fracture (n .125 - 55.5%) and gingival recession (n.118 - 52%). About risk of lingual abscesses, 141 gave positive feedback (62%) while only 56 for diastema and 57 for hyper salivation (about 25% of the overall data). It is interesting to note the differences in awareness of oral piercing complications between males and females (Fig. 2). Males were more prepared with regard endocarditis, Ludwig angina, gingival infection, tooth fracture or chipping, diastema and hyper salivation. Females, on the other hand, were more informed about temporary and permanent paralysis and allergic reactions to the materials used. The knowledge about risk of gingival recession and lingual abscesses have no detectable differences between the two groups.The questionnaire also explored other fields of knowledge, in particular risks of cross infection. The knowledge of males and females about HIV, HAV and HBV are almost similar, but as far as the knowledge of HCV boys were more aware, while awareness of HSV was higher among females (Fig. 3). As already explained in the Materials and Methods section, to test some variables related to the practice of piercing the students with oral or perioral piercing were assessed. More than 50% of teens surveyed was found to have a piercing that was 121 on 225 divided as follows: 46 with lip piercing, 35 with tongue piercing, 13 with labial frenulum piercing, 16 with lingual frenulum piercing, 11 with pierced cheek piercing. The piercing wearers were asked to answer additional questions about the experience on the day they were pierced. It is interesting to note the different site distribution of piercing between males and females (Fig. 4). Lips and the lingual frenulum piercings turn out to be fashionable among males, while tongue and cheek piercings the language and the cheeks were more preferred by females. Lingual frenulum piercing appeared to be of equal interest between males and females.

Other questions also focused on the moment of first wearing piercing (Fig. 5).

Only 17% (21 teens) checked the piercer license and only 18% (22 adolescents) sterilization and disinfection of the materials used. 53.7% (65 teens) did not received explanations about the risks associated with piercing. With regard to the maintenance mode of the piercing, in 61.2% of cases (74 adolescents) it has been suggested to rinse with mouthwash, while in 17% of cases ( 21 teens) to brush the piercing. In addition, 33% (40 adolescents) of the respondents had suggestions about the use of antibiotic creams. The post piercing specialist visits have been suggested only in 7% of cases (9 teens). Therefore females were found to be more attentive to the control of material disinfection compared to males. It also appeared that the explanations of risks and mode of piercing maintaining (rinsing with mouthwash, brushing piercing and using antibiotic creams) have been provided mostly to females. The control of the piercer license and advice on the appropriateness of a check post-piercing were extremely low throughout the sample interviewed. At the end of the questionnaire compilation all participants received a brochure explaining risks and advice to follow for maintaining a good oral hygiene.

## Discussion

Oral piercing has become common among young adults during the recent decade (10). However, piercing is not without risks (11). Descriptions of both immediate and delayed complications appear in many case reports (12,13). More than 50% of teens surveyed was found to wear a piercing. The most relevant data were received by the students with oral piercings. Adolescents that were questioned in this study were in general not exactly aware of all the complications that could be expected after oral piercing. Oral piercings have a high potential for infectious complications, because they invade the subcutaneous tissues and disrupt mucosal integrity, and place a foreign body within the wound. The wound originated from the insertion of the jewelry can allow various and numerous microorganisms that normally inhabit the oral cavity to enter the bloodstream and develop infections in other organs such as heart liver or brain (14,15)

The results regarding cross-infection with HIV and HSV have been positive in the entire sample of respondents, while poor knowledge resulted about the risk of hepatitis from HCV, HAV and HBV (approximately 74.77 % was not aware of). It was then verified awareness related to the risks of performing a piercing and the results have been disappointing, except for temporary paralysis, chipping or fracture tooth and lingual abscesses, which were found to be widely recognized. The general lack of awareness of the different risks of oral piercing could be attributed to piercers often not licensed, who are not aware of the possible complications of oral piecing or prefer not to tell the patients so as not to scare them (16) off. Very few of the teens surveyed (18%) checked the effective piercing sterilization at the time of performing and only 7.69% of them received the advice of a post-piercing dentist checkup. The same universal precautions for preventing transmission of infectious agents must be applied to piercing as to any other invasive procedure. It is recommended that people who want to wear an oral piercing look for a licensed piercer who is familiar with infection control measures, including the use of sterile single-use needles, disposable gloves and instruments, and an autoclave for inter-patient sterilization (3). It’s also important that the piercer make an appropriate jewellery material selection in order to avoid allergy. According to the Association of Professional Piercers, the materials to be used for piercing should be stainless steel or titanium, 14K gold or higher, platinum or PTFE (Teflon) (17).

The distribution of the brochure was useful to increase awareness of risks and maintenance mode. The piercing should be removed daily and cleaned and brushed thoroughly to maintain good oral

hygiene (12). Oral health care might be an important tool to minimize early and late postpiercing complications.

## Conclusion

The general lack of awareness of complications and maintenance mode related to oral piercing needs to be addressed by some education programs performed at school and by dentists. When a patient with an oral piercing arrives at a dental office for a periodic check-up, the structures surrounding oral and perioral piercings should be evaluated as a part of the oral check-up. Oral piercing wearer should also be informed of possible complications and piercing maintenance.
